# A Promising Approach to Ultra‐Flexible 1 Ah Lithium–Sulfur Batteries Using Oxygen‐Functionalized Single‐Walled Carbon Nanotubes

**DOI:** 10.1002/advs.202406536

**Published:** 2024-12-04

**Authors:** Junyoung Heo, Jeong‐Won Hong, Ha Won Gu, Junghwan Sung, Dong‐Hee Kim, Jung Hoon Kim, Sung Kang, You‐Jin Lee, Hye Young Choi, Doohun Kim, Kang‐Jun Baeg, Joong Tark Han, Jun‐Woo Park

**Affiliations:** ^1^ Next‐Generation Battery Research Center Korea Electrotechnology Research Institute (KERI) 12, Jeongiui‐gil, Seongsan‐gu, Changwon‐si Gyeongsangnam‐do 51543 Republic of Korea; ^2^ Department of Electro‐Functionality Materials Engineering University of Science and Technology (UST) 217, Gajeong‐ro, Yuseong‐gu Daejeon 34113 Republic of Korea; ^3^ Nano Hybrid Technology Research Center Korea Electrotechnology Research Institute (KERI) 12, Jeongiui‐gil, Seongsan‐gu, Changwon‐si Gyeongsangnam‐do 51543 Republic of Korea; ^4^ Analysis and Assessment Research Center Research Institute of Industrial Science & Technology (RIST) 67 Cheongam‐ro, Nam‐gu Pohang‐si 37673 Republic of Korea; ^5^ Major of Semiconductor Engineering Pukyong National University 45, Yongso‐ro, Nam‐gu Busan 48513 Republic of Korea

**Keywords:** lithium polysulfides, lithium–sulfur batteries, shuttle effect, single‐walled carbon nanotubes, sulfur composite cathodes

## Abstract

Lithium–sulfur (Li‐S) batteries represent a promising solution for achieving high energy densities exceeding 500 Wh kg^−1^, leveraging cathode materials with theoretical energy densities up to 2600 Wh kg^−1^. These batteries are also cost‐effective, abundant, and environment‐friendly. In this study, an innovative approach is proposed utilizing highly oxidized single‐walled carbon nanotubes (Ox‐SWCNTs) as a conductive fibrous scaffold and functional interlayer in sulfur cathodes and separators, respectively, to demonstrate large‐area and ultra‐flexible Li‐S batteries with enhanced energy density. The free‐standing sulfur cathodes in the Li‐S cells exhibit high energy density maintaining 806 mAh g^−1^ even after 100 charge‐discharge cycles. Additionally, oxygen‐containing functional groups on the SWCNTs significantly improve electrochemical performance by promoting the adsorption of lithium polysulfides. Employing Ox‐SWCNTs in both cathodes and interlayers, the study achieves high‐capacity Li‐S pouch cells that consistently deliver a capacity of 1.06 Ah and a high energy density of 909 mAh g^−1^ over 50 charge‐discharge cycles. This strategy not only significantly enhances the electrochemical performance of Li‐S batteries but also maintains excellent mechanical flexibility under severe deformation, positioning this Ox‐SWCNT‐based architecture as a viable, light‐weight, and ultra‐flexible energy storage solution suitable for commercializing rechargeable Li‐S batteries.

## Graphical abstract

The successful development of a large‐size pouch‐type Li‐S cell comprising six stacks, each containing ten cathodes with dimensions of 50 × 60 mm^2^. The cell features integration of highly oxidized single‐walled carbon nanotubes with sulfur electrodes and functional interlayer on a membrane, achieving both high energy density and the ability to sustain capacities over 1 Ah through extended charge‐discharge cycles.

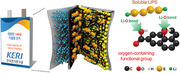

## 1 Introduction

Recently, there has been an increasing demand for sustainable technologies and products. In this realm, products that incorporate rechargeable batteries, such as electric vehicles and energy storage systems, play a pivotal role in reducing carbon emissions and fostering sustainable energy practices and have gained widespread popularity. Lithium‐ion batteries (LIBs) dominate the market due to their many advantages, including high energy density, high charge and discharge efficiency, and scalability.^[^ ^1^ ^]^ However, they exhibit several limitations in terms of stability and have a relatively low energy density compared to various next‐generation batteries that are being considered as alternatives.^[^ ^2^ ^]^ Lithium–sulfur (Li‐S) batteries are emerging as a compelling alternative to the prevalent LIBs, catering to the rapidly growing energy demand.^[^ ^3^, ^4^, ^5^, ^6^, ^7^ ^]^ The Li‐S systems, which combine abundant sulfur with metallic lithium, potentially offer an energy density nearly five times greater at approximately one‐third the cost compared to LIBs.^[^ ^8^ ^]^ Particularly, the cathode materials in Li‐S batteries, noted for their exceptionally high theoretical energy density (up to 2600 Wh kg^−1^), cost‐effectiveness, abundant availability, and environmental compatibility, present a viable solution for achieving Li‐S batteries with an energy density exceeding 500 Wh kg^−1^.^[^ ^2^, ^9^, ^10^, ^11^, ^12^ ^]^

There are three main barriers to the widespread implementation of Li‐S batteries. Firstly, sulfur electrochemistry is constrained by the insulating natures of active materials and volumetric changes during the charge‐discharge cycles, resulting in weakened electrode integrity during cycling.^[^ ^8^ ^]^ Secondly, employing reactive lithium metal can lead to severe electrolyte decomposition and dendrite formation, causing serious safety issues such as internal electrical failure, heat accumulation, and gas release.^[^ ^13^ ^]^ The most critical challenge, however, lies in the behavior and presence of the intermediate species, polysulfide (PS), within the complex sulfur electrochemistry. This aspect distinctively characterizes Li‐S battery systems. PS intermediates tend to dissolve in the organic electrolyte, leading to a substantial loss of active material. This dissolution contributes to severe Coulombic inefficiency, corrosion of the lithium metal anode, and, consequently, accelerated cell failure.^[^ ^14^, ^15^ ^]^ Therefore, the effective management of PS shuttle behavior is imperative for enhancing the electrochemical performance and facilitating the practical application of Li‐S batteries.^[^ ^3^ ^]^

Substantial advancements and enhancements have been realized over the previous decade to address the aforementioned challenges. In particular, among the various components of Li‐S batteries, the element sulfur, as the active material in the cathode, is the most critical because it is closely related to the battery structure and determines the outstanding electrochemical performance. Consequently, the sulfur cathode has been the focus of rigorous research efforts.^[^ ^16^ ^]^ However, the inherent properties of sulfur as an insulator make it unsuitable for direct use as an electrochemically active material due to its low intrinsic electrical conductivity.^[^ ^17^ ^]^ To mitigate this limitation, sulfur is frequently combined with diverse conductive nano/micro materials, including porous carbon,^[^ ^18^, ^19^, ^20^ ^]^ graphene,^[^ ^21^, ^22^, ^23^, ^24^, ^25^, ^26^ ^]^ and carbon nanotubes.^[^ ^27^, ^28^, ^29^ ^]^ Despite these strategies, the conventional conductive porous structure remains insufficient in simultaneously achieving the high sulfur(S) loading and electrical conduction pathways, and fully capturing dissolved polysulfides within the electrolyte due to weak interactions with carbon‐based materials. This inadequacy leads to a decrease in electrochemical performance, notably manifesting as accelerated degradation during charge‐discharge cycles. In addressing these drawbacks, employing highly conductive carbon nanomaterials incorporated with functional groups capable of interacting with PS provides a promising approach.^[^ ^16^, ^30^, ^31^ ^]^ In this context, single‐walled carbon nanotubes (SWCNTs) stand out as a viable option because they offer the advantage of forming highly conductive networks while requiring only a minimal amount of SWCNTs in electrodes.^[^ ^4^, ^30^ ^]^ However, significant challenges remain in controlling the oxidation of SWCNTs to prevent fragmentation and deterioration of electrical conduction, as well as in minimizing bundle size, which are crucial steps for their integration into next‐generation energy storage systems.

In this study, we present two primary concepts using SWCNTs to enhance the electrochemical performance of Li‐S cells. First, we propose a Li‐S battery featuring a free‐standing sulfur cathode with high S loading, composed of an oxidized SWCNT‐based conductive fibrous structure. This cathode facilitates fast electrochemical reactions via an efficient electrical conduction pathway, provided by its porous structure with high wettability for the liquid electrolyte. Additionally, strategically designed SWCNTs containing oxygen functional groups play a crucial role in preventing lithium polysulfide (LiPS) precipitation. They do so by forming smaller bundles and obstructing the diffusion of Li_2_S_x_ toward the anode through the formation of Li‐O bonds. With these multifunctional benefits, we achieved high‐capacity Li‐S batteries that maintained a capacity of 806 mAh g^−1^ even after 100 cycles. Second, we developed a commercial membrane coated with oxidized SWCNT interlayers. This carbon‐based interlayer employs a dual trapping mechanism to effectively mitigate the diffusion of Li_2_S_x_ toward the anode. Finally, large‐area, ultra‐flexible pouch‐type Li‐S cells with a capacity exceeding 1 Ah were successfully fabricated using highly oxygen‐functionalized conductive SWCNTs incorporated into both the cathode and the interlayer.

## 2 Results and Discussion

### 2.1 High‐Loading Sulfur Cathodes Based on Oxygen‐Functionalized SWCNTs

**Figure 1:**
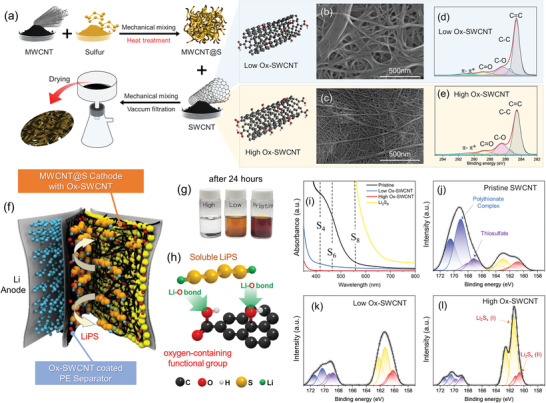
Overview of materials and analysis methods: a) diagram illustrating the fabrication processes for synthesizing MWCNT@S powder and the preparation of an oxidized SWCNT‐based sulfur cathode. SEM images of b) Low Ox‐SWCNTs and c) high Ox‐SWCNTs. XPS profiles of d) Low Ox‐SWCNTs and e) high Ox‐SWCNTs. f) Schematic of Li‐S battery structure alongside the shuttling behavior of LiPS. g) Photograph of the DOL/DME solution maintained at room temperature for 24 h after adding a Li_2_S_8_ solution and various types of SWCNTs (Pristine SWCNTs, Low Ox‐SWCNTs, and High Ox‐SWCNTs). h) Schematic of the chemical interactions between soluble LiPS and oxygen functional groups in SWCNTs. i) UV–vis absorption profiles of the Li_2_S_8_ solutions after adding various types of SWCNTs. XPS spectra of S2p in three types of SWCNTs: j) pristine SWCNTs, k) low Ox‐SWCNTs, and l) high Ox‐SWCNTs after mixing with Li_2_S_8_.

**Figure** **1**a illustrates the step‐by‐step process for fabricating a free‐standing sulfur cathode with carbon nanotubes (CNTs). In brief, Multi‐walled carbon nanotubes(MWCNTs) and S powder (MWCNT@S) were prepared by mixing S and pristine MWCNTs, followed by high‐energy ball milling and 2‐hour thermal treatment at 155 °C to achieve melt‐diffusion of elemental S into CNT network structure.^[^ ^32^, ^33^ ^]^ Mechanical ball milling was then employed to mix an aqueous suspension of CNTs with powder‐type single‐walled carbon nanotubes (SWCNTs) and MWCNT@S. After vacuum filtration, the composite S cathodes were finally prepared.^[^ ^34^, ^35^, ^36^ ^]^ Both MWCNTs and SWCNTs were used as conductive components in the free‐standing and high‐loading S cathodes. Since MWCNTs are easier to prepare and less expensive than SWCNTs, they facilitate higher active material loading. Meanwhile, SWCNTs primarily form a highly conductive 3D network, enabling high discharge rates even at high S‐loadings.^[^ ^16^, ^33^, ^37^ ^]^ Additionally, the SWCNTs were functionalized with different bundle sizes and amounts of oxidative groups to serve as a conductive additive interacting with sulfur. In this study, three different aqueous suspensions containing SWCNTs with varying degrees of oxidation were utilized. The bundle structure and oxidation level of SWCNTs were controlled by varying the amount of oxidant, KMnO_4_, and reaction time. Before oxidation, the SWCNTs in H_2_SO_4_ were pre‐debundled by adding K_2_SO_4_ as an intercalant, because the K^+^ and SO_4_^2−^ ions can be easily intercalated into graphitic structures in acidic conditions.^[^ ^38^, ^39^ ^]^ The amount of oxidant can control the final bundle size as they can also be intercalated into the SWCNT bundle during oxidation. To obtain large bundles with low oxidation levels of SWCNTs (referred to as Low Ox‐SWCNTs), SWCNTs were oxidized using a 1:0.5 weight ratio of SWCNT to KMnO_4_ and reacted for 0.5 hours. For comparison, small bundle‐sized SWCNTs with high oxidation levels (referred to as High Ox‐SWCNTs) were fabricated by increasing the amount of KMnO_4_ to a 1:1 weight ratio and extending the reaction time to 1 hour. During oxidation, KMnO_4_ molecules oxidize the end of SWCNTs, which can promote the debundling of SWCNT bundles through reaction time.

Field emission scanning electron microscope (FE‐SEM) images in Figure 1b,c show the Low Ox‐SWCNTs and High Ox‐SWCNTs, respectively. When analyzing the SEM images, it was verified that Low Ox‐SWCNTs exhibit larger bundles exceeding 100 nm in diameter compared to High Ox‐SWCNTs, which exhibited smaller and more densely packed bundles. This observation confirmed that the SWCNT bundle size could be readily controlled by changing the quantity of KMnO_4_ and the reaction time. The C1s X‐ray photoemission spectroscopy (XPS) data of the oxidized SWCNTs indicated that carboxylic acid (288.7 eV) and hydroxyl (286.3 eV) groups were successfully introduced on the SWCNT surface and/or at the end of the nanotubes (Figure 1d,e). By increasing the amount of oxidant, the area percentage of C─O bond increased from 19.1% to 23.7% without a significant change in the percentage of C = O bonds (8%), corresponding to TGA data (Figure S1, Supporting Information). This suggested that hydroxyl groups were introduced on the SWCNT surface during the debundling and oxidation processes. Raman spectra of the Low Ox‐SWCNTs and High Ox‐SWCNTs also show the generation of defects on the SWCNT surface by oxidation (Figure S2, Supporting Information).

Figure 1f shows the schematic of the Li‐S battery structure with S cathodes made from three different oxygen‐functionalized SWCNTs. UV–vis absorption spectroscopy and XPS studies were carried out to investigate the interaction effect between chemically functionalized SWCNTs and LiPS. The preparation process begins with crafting a 0.01 M Li_2_S_8_ solution in 1,3‐dioxolane/1,2‐dimethoxyethane (DOL/DME) (1/1 v/v). Ox‐SWCNTs with different oxidation levels were immersed in 5 mL DOL/DME solutions, followed by injecting an equivalent volume of the prepared Li_2_S_8_ solution. After the Li_2_S_8_ solution was injected into the cathodes containing Ox‐SWCNTs, the solution initially exhibited a common dark brown color. However, the color of the solution with the Low Ox‐SWCNT and High Ox‐SWCNT cathodes gradually changed to lighter brown after 12 hours. In contrast, the solution with the pristine SWCNT maintained its dark brown color. Moreover, after 24 hours, the solution with the High Ox‐SWCNT cathode became transparent, while those with the Low Ox‐SWCNT retained a light brown color, as seen in Figure 1g. Visual observation confirmed that cathodes using High Ox‐SWCNTs, characterized by a higher content of oxygen‐functional groups, absorb a more significant amount of LiPS by forming a Li‐O bond between oxygen‐containing functional groups on SWCNTs and LiPS. From the UV–vis absorption spectroscopy of Li_2_S_8_ solutions (Figure 1i), the pure LiPS solution with its dark brown color exhibited an absorption peak ≈560 nm wavelength, indicating an association with S_8_. After 24 hours, the Li_2_S_8_ solution with pristine SWCNTs showed weak absorption ≈560 nm and sharply rising absorption ≈420 and 470 nm due to uncaptured low‐order polysulfides, such as S_4_ and S_6_. In contrast, those PS peaks for both High Ox‐SWCNT and Low Ox‐SWCNT cathodes are significantly lower. This indicates that pristine SWCNT cathodes hardly capture the PS, while High Ox‐SWCNT and Low Ox‐SWCNT cathodes effectively prevent the escape of polysulfides. The adsorption phenomenon was further investigated through XPS spectra analysis (Figure 1j–l). The S2p spectra of the High Ox‐SWCNT exhibited characteristic peaks at the binding energy of 161.3 and 160.6 eV, which indicate higher‐order LiPS (Li_2_S_x_ (I)) and lower‐order LiPS (Li_2_S_x_ (II)), respectively. XPS peaks at 170.4 and 168.8 eV correspond to thiosulfate and polysulfate complexes, respectively. Notably, in the High Ox‐SWCNT cathode, the intensity of the Li_2_S_x_ (I) peak was the highest. In contrast, the same PS peaks in the Low Ox‐SWCNT and pristine SWCNT cathodes exhibited relatively lower intensities, suggesting significant absorption of higher‐order PS by oxygen functional groups. Because the High Ox‐SWCNTs have a higher content of oxygen‐functional groups, a smaller diameter, and a densely packed bundle structure, they absorb a significant amount of LiPS by forming Li‐O bonds. Additionally, their nanoporous structure enhances interaction by increasing the number of active sites and physically trapping LiPS in the sulfur cathodes.

**Figure 2:**
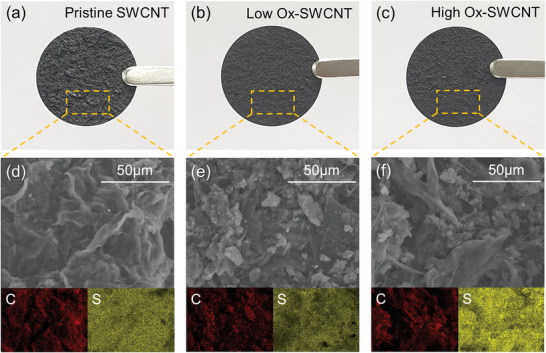
Digital camera images and analysis of CNT‐based sulfur cathodes: a) digital photographs showing free‐standing pristine SWCNTs, b) one using Low Ox‐SWCNTs, and c) one using High Ox‐SWCNTs. SEM and EDS mapping images of the CNT‐based sulfur cathodes featuring various types of SWCNTs: d) pristine SWCNTs, e) low Ox‐SWCNTs, and f) high Ox‐SWCNTs.

The as‐prepared MWCNT@S was combined with various SWCNTs (Pristine, Low Ox‐SWCNTs, or High Ox‐SWCNTs), in which the SWCNT powder was dispersed in H_2_O through ball‐milling for 10 minutes. Consequently, free‐standing S cathodes (thickness = 150–350 µm, depending on the loading value)) were prepared by vacuum filtration and drying at 60 °C for 12 hours in a vacuum oven. **Figure** **2**a–c show digital camera images of sulfur electrodes fabricated using either pristine SWCNTs or those that have been oxygen‐functionalized. Pristine SWCNTs have superior electrical characteristics and distinctive thermal and physicochemical properties. However, they cannot exist independently due to the strong Van der Waals forces that cause them to form bundles, leading to the partially agglomerated shapes observed in Figure 2a. Conversely, Ox‐SWCNTs in Figure 2b,c exhibited comparatively uniform surfaces. This uniformity was mainly attributed to the excellent dispersion of Ox‐SWCNTs in aqueous solution, achieved through a specific pretreatment process. The SEM images in Figure 2d–f, along with elemental mapping results obtained from energy‐dispersive X‐ray spectroscopy (EDS), also demonstrated that sulfur electrodes developed from SWCNTs with oxygen‐containing functional groups exhibited a well‐mixed and evenly distributed carbon and sulfur composition unlike those based on Pristine SWCNTs. Additionally, the EDS mapping results for oxygen elements are provided in Figure S3 (Supporting Information).

**Figure 3:**
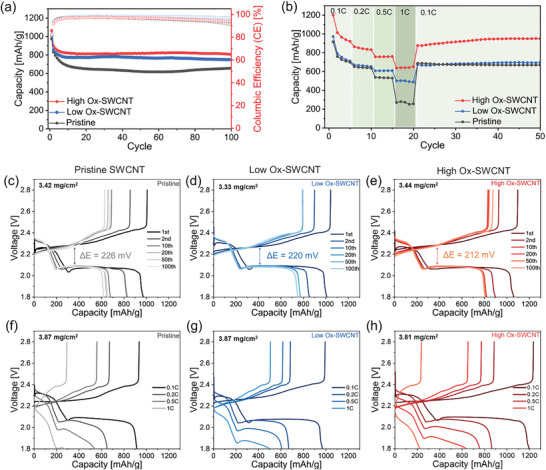
Electrochemical performance of Li‐S cells with SWCNT‐based sulfur cathodes. a) Discharge capacity and Coulombic efficiency (CE) of Li‐S cells employing sulfur cathodes with different types of SWCNTs. b) Cycling performance profiles at varying C‐rates from 0.1C to 1.0C. c–e) Voltage profiles for Li‐S cells with Pristine SWCNT (c), low Ox‐SWCNTs (d), and high Ox‐SWCNTs (e) at 0.1C. f,g) Voltage profiles at various C‐rates (0.1, 0.2, 0.5, and 1C) for the Li‐S cells with various types of SWCNT‐based sulfur cathodes.

The oxygen‐functionalized SWCNTs were incorporated into the sulfur cathodes of Li‐S batteries, and combined with commercial polyethylene (PE) to evaluate their electrochemical performance. As depicted in **Figure** **3**a, all Li‐S cells exhibited similar initial discharge capacities, ≈1000 mAh g^−1^ at 0.1C. However, Li‐S batteries with pristine SWCNT‐based sulfur cathodes demonstrated relatively low capacities of 656 mAh g^−1^ after 100 charge/discharge cycles, corresponding to a retention of ≈67% of the initial capacity. In contrast, Li‐S batteries utilizing Ox‐SWCNT cathodes showed superior retention properties after the same number of cycles. Interestingly, an increase in the amount of oxygen‐containing functional groups on the SWCNTs correlated with higher discharge capacities in Li‐S cells. Specifically, cells equipped with Low Ox‐SWCNTs and High Ox‐SWCNTs electrodes exhibited capacities of ≈749 and 805 mAh g^−1^ after 100 cycles, respectively. This suggested that amount of oxygen functional groups on the SWCNT surface significantly enhanced the electrochemical stability of Li‐S cells by capturing polysulfides within a conductive fibrous network and inhibiting their shuttling, compared to the reference electrodes without functional groups (Pristine SWCNTs). Figure 3b illustrates the cycling performance of Li‐S batteries as a function of C‐rates with various sulfur cathodes containing different concentrations of oxygen functional groups on SWCNTs. Notably, the pristine SWCNTs displayed a sharp decrease in discharge capacities with increasing current densities, delivering only 270 mAh g^−1^ at 1C. Consequently, our Li‐S batteries based on High Ox‐SWCNT electrodes maintained remarkably high capacities, exceeding 630 mAh g^−1^ at 1C.

Figure 3c–e illustrate the charge‐discharge profiles of Li‐S cells utilizing Pristine, Low Ox‐ and High Ox‐SWCNTs electrodes across the 1st, 2nd, 10th, 20th, 50th, and 100th cycles at 0.1C. The initial charge curves within these voltage profiles showed similar potential barriers, suggesting the absence of non‐conductive Li_2_S_2_ and Li_2_S species on the electrode surfaces. This is attributed to the high electrical conductivity of SWCNTs, which facilitates rapid charge and mass transport, leading to swift electrokinetic reactions. However, when comparing the average overpotentials, electrodes with pristine SWCNT demonstrated a relatively large overpotential of 226 mV. In contrast, those containing High Ox‐SWCNTs exhibited a smaller overpotential of ≈212 mV, indicative of a lower nucleation potential. Further details regarding the overpotential differences can be found in Figure S4a (Supporting Information), which shows the voltage profile of the second charge‐discharge highlighting the overpotential at 50% of the total discharge capacity. This reduction in overpotential suggests that a higher concentration of oxygen functional groups enhances the conversion efficiency of long‐chain polysulfides to Li_2_S, thereby improving the solid‐solid reduction kinetics. Moreover, the second plateau in the Li‐S cell voltage profiles, which corresponds to the formation of short‐chain polysulfides, highlights slow kinetic reactions. As depicted in Figure 3f, this plateau region was not distinct in cells with Pristine SWCNT electrodes at high C‐rates. Conversely, cells with both Low Ox‐ and High Ox‐SWCNT electrodes exhibited clear, well‐defined plateau shapes even at elevated current densities. Despite the high conductivity of SWCNTs, the transformation to lower‐order polysulfides was hindered at high current densities in the pristine case due to slow reaction kinetics. Thus, introducing oxygen functional groups on the SWCNT surface likely increases the number of anchoring sites available for chemical interaction with LiPS and enhances Li^+^ mobility, even under conditions of higher current density.

### 2.2 Ox‐SWCNTs Functional Interlayer for Separators

**Figure 4:**
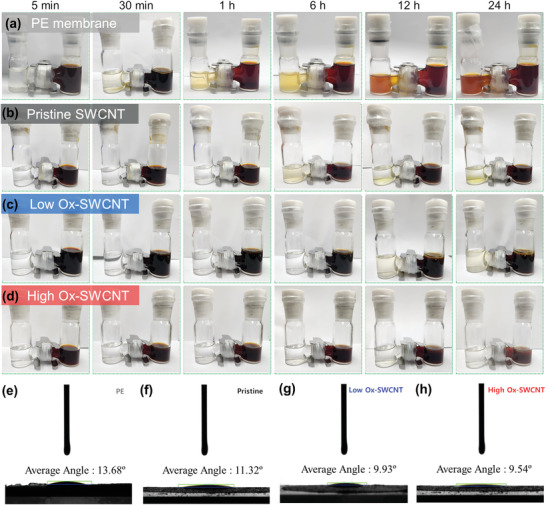
Visualization of diffusion properties and wettability assessments of Li_2_S_x_ solutions through various separators; a) commercial PE membrane, and PE membrane coated with b) pristine SWCNTs, c) PE membrane coated with Low Ox‐SWCNTs, and d) PE membrane coated with High Ox‐SWCNTs interlayers. Contact angle measurements of the liquid electrolyte on e) bare PE membrane, f) pristine SWCNT‐coated PE membrane, g) low Ox‐SWCNT‐coated PE membrane, and h) high Ox‐SWCNT‐coated PE membrane.

The principal challenge in commercializing Li‐S batteries is the shuttle effect, characterized by the migration of soluble LiPS through permeable separators. This issue can be mitigated by incorporating functional interlayers, such as nanocarbons, activated carbons, or their blends, onto commercial membranes. The Ox‐SWCNTs appear particularly promising as interlayers due to their ability to chemically interact with LiPS, forming Li‐O bonds, and physically trap these species within the micropores of fibrous CNT networks. In this study, we evaluate the effectiveness of Ox‐SWCNT interlayers in mitigating the shuttle effect as a function of the density of oxygen‐containing functional groups, using a commercial PE separator. **Figure** **4**a–d depict a simple H‐type cell experimental setup to visually access the suppression of the shuttle effect. A standard PE separator was positioned between a polysulfide solution (0.01 M Li_2_S_8_ in a 1/1 v/v DOL/DME solution) on the right side and a pure solvent mixture (DOL/DME at a 1:1 v/v ratio) on the left side, with the SWCNT‐coated surface facing the Li_2_S_8_ solution. Over 24 hours, the solvent mixture on the left side transitioned from pale yellow to dark brown, indicating LiPS diffusion through a single PE membrane (Figure 4a). In contrast, membranes coated with Pristine SWCNT interlayers maintained a lighter yellow color, suggesting partial suppression of LiPS diffusion primarily through physical entrapment within the porous CNT structure, albeit with limited chemical interaction (Figure 4b). Introduction of oxygen‐functional groups on the SWCNT surface markedly improved this behavior; a membrane with Low Ox‐SWCNT retained a subtle yellow hue (Figure 4c), while one with High Ox‐SWCNTs maintained its initial transparency (Figure 4d). This enhanced effectiveness in mitigating LiPS diffusion is attributed to the increased capacity of oxygen‐enriched functional groups to form a greater number of Li‐O bonds, facilitating stronger chemical interactions.

Furthermore, compatibility and intimate contact between the electrolytes and electrodes are crucial for achieving superior electrochemical performance in Li‐S batteries. The introduction of substantial oxygen functional groups into the carbon intermediate layer increases its polarity and surface energy, thereby enhancing the wettability of PE membrane. This enhancement facilitates more efficient infiltration of liquid electrolytes into the sulfur cathodes. As shown in Figure 4e–h, we conducted contact angle measurements to evaluate the wettability of SWCNT‐based intermediate layers. The untreated bare PE, with its non‐polar surface characteristics, displayed a relatively high contact angle of 13.68°. In contrast, a Pristine SWCNT‐coated membrane showed a reduced contact angle of 11.32°. Moreover, the incorporation of oxygen‐containing functional groups into the SWCNTs further decreased the contact angle to 9.93° and 9.54° for Low Ox‐ and High Ox‐SWCNT, respectively. These results confirm that the significant presence of oxygen functional groups in the carbon intermediate layer substantially improves the wettability and compatibility of the PE membrane with the electrolytes.

**Figure 5:**
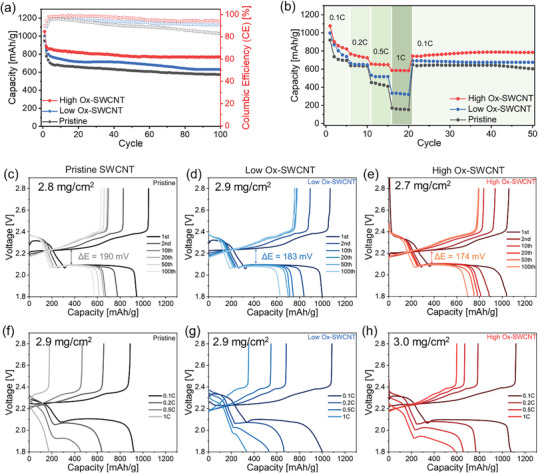
Electrochemical performance of Li‐S batteries with SWCNT interlayers: a) discharge capacities and Coulombic efficiency (CE) of the Li‐S cells employing various SWCNT interlayers on a PE membrane. b) Cycling performance profiles across different C‐rates (from 0.1 C to 1.0 C) for Li‐S cells with different SWCNT interlayers. Voltage profiles for Li‐S cells with CNT‐based interlayers on a PE separator, featuring c,f) pristine SWCNT, d,g) Low Ox‐SWCNTs, and e,h) High Ox‐SWCNTs. Profiles (c–e) represent 100 charge/discharge cycles at a fixed current density, at 0.1C, and (f‐h) span various C‐rates of 0.1, 0.2, 0.5, and 1C.

We investigated the electrochemical performance of Li‐S batteries to assess the effect of SWCNT interlayers enriched with oxygen functional groups. In the previous section, where we discussed Ox‐SWCNT‐incorporated sulfur cathodes, we utilized a commercial PE separator to isolate and evaluate the cathode performance when employing oxidized SWCNTs. In this section, we employed pristine SWCNT‐based cathodes devoid of oxygen functional groups to investigate the interlayer properties of Ox‐SWCNTs with varying oxygen functional group concentrations. **Figure** **5**a shows the long‐term cycling characteristics of Li‐S batteries, measured over 100 cycles at a 0.1C rate, with different SWCNT interlayers on a PE membrane. Initially, all Li‐S cells exhibited discharge capacities ≈900 mAh g^−1^. However, cells with pristine SWCNT interlayers demonstrated a significant capacity decline to 574 mAh g^−1^, with Coulombic efficiencies (CE) of only 82% after 100 cycles. Conversely, Li‐S cells incorporating High Ox‐SWCNT and Low Ox‐SWCNT interlayers retained higher capacities of 695 and 628 mAh g^−1^, respectively, after 100 cycles, achieving CEs exceeding 94% and 90%. These results suggested that a higher concentration of oxygen functional groups on SWCNTs enhances capacity retention and mitigates irreversible capacity loss by suppressing the polysulfide shuttle effect.

To further understand the impact of different oxygen functional group concentrations under high current density conditions, we examined the cycling performance across several C‐rates (Figure 5b). While the initial electrochemical performances at a low C‐rate were comparable across configurations, significant differences emerged at higher rates exceeding 0.2C. Notably, cells with High Ox‐SWCNT interlayers reached the highest capacity of 585 mAh g^−1^ at 1C, whereas those with Low Ox‐SWCNT and pristine SWCNT interlayers displayed markedly lower capacities of 334 and 170 mAh g^−1^, respectively, under the same conditions. Voltage profiles measured over 100 cycles (Figure 5c–e) further demonstrated an average overvoltage of ≈174 mV for the High Ox‐SWCNT interlayer, compared to 183 and 190 mV for the Low Ox‐SWCNT and pristine SWCNT interlayers, respectively. These findings underscore the efficacy of oxygen functional groups on the SWCNT surface in inhibiting the LiPS shuttle phenomenon. The voltage profiles of the second charge‐discharge cycle for the High Ox‐SWCNT, Low Ox‐SWCNT, and pristine SWCNT interlayers are compared, illustrating the overvoltage differences at the point corresponding to 50% of the total discharge capacity, as shown in Figure S4b (Supporting Information). Further analysis of voltage profiles at varied C‐rates (Figure 5f–h) revealed that Li‐S cells with High Ox‐SWCNT interlayers exhibited a broad second discharge plateau, indicative of the formation of low‐order polysulfides even at 1C, thereby maintaining high capacities. In contrast, cells with Low Ox‐SWCNT and pristine SWCNT interlayers exhibited a narrower plateau region due to the shuttle phenomenon. Consequently, it was confirmed that increased oxygen functional group concentrations on SWCNT interlayers enhanced both capacity and stability under high current density conditions.

**Figure 6:**
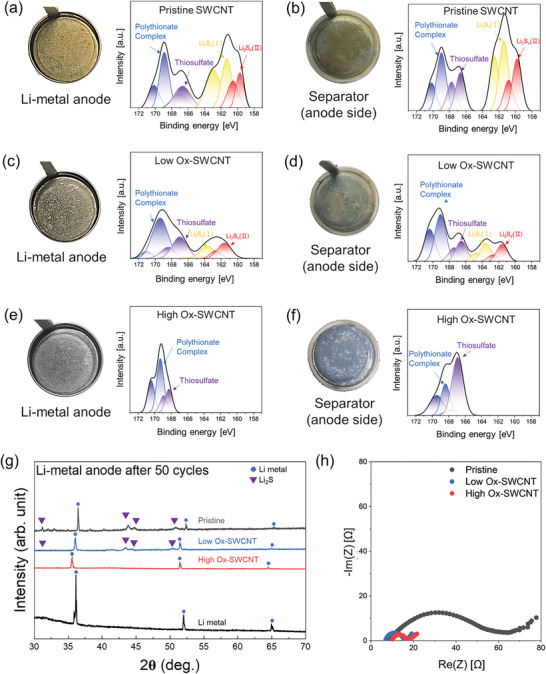
a–f) Digital photographs and corresponding XPS profiles of disassembled Li‐S cells after 50 charge/discharge cycles. Panels (a,c,e) show Li‐metal anode, and (b,d,f) depict the anode side of the PE separator, with varying degrees of oxygen functional groups introduced on SWCNT. g) XRD patterns of pristine Li‐metal and Li‐metal anode after 50 cycles with pristine SWCNTs, low Ox‐SWCNTs, and high Ox‐SWCNTs interlayers. h) EIS Nyquist plots representing impedance spectra after the 100th discharge cycle, illustrating the effects of different SWCNT interlayers on interfacial resistance.

**Figure** **6**a–f show digital camera images and corresponding XPS analyses of chemical species for the disassembled Li‐S cells after 50 cycles, using different types of SWCNT interlayers on a PE separator. Cells with pristine SWCNT and Low Ox‐SWCNT interlayers showed undesired color changes and uneven metal surfaces due to the shuttle phenomena of Li_2_S_x_. In contrast, both the anode and separator (anode side) in cells with the High Ox‐SWCNT interlayer retained their original color and exhibited clean surfaces without Li_2_S passivation layer deposition. This observation confirms that oxygen functional groups on the SWCNT surface can effectively inhibit polysulfide diffusion through the membranes. XPS analysis revealed that surfaces of the Li‐metal anode and separator (anode side) in cells with pristine SWCNT interlayers showed intense peaks at 159.6 eV and 163.1 eV, assigned to high‐order Li_2_S_x_ (II) and low‐order Li_2_S_x_ (I), respectively. The inability of the pristine SWCNT interlayer to suppress polysulfide diffusion resulted in the accumulation of insoluble polysulfides on the anode, causing discoloration and a rough electrode surface. After introducing oxygen functional groups on the SWCNT interlayer, the intensity of these polysulfide peaks decreased with increasing oxidation levels. To further investigate the stability of oxygen functional groups after long‐term cycling, XPS analysis was conducted on the cathodes after 50 cycles, as shown in Figure S5 (Supporting Information). The results confirm that oxygen functional groups, such as C═O, C─O, and Li‐O bonds, remain present even after extensive cycling, with corresponding binding energies at 532.6, 531.4, and 530.3 eV, respectively. Notably, the High Ox‐SWCNT and Low Ox‐SWCNT samples exhibited stronger Li‐O bond intensities compared to the pristine sample, indicating that oxygen functional groups continue to enhance electrochemical stability by chemically binding lithium polysulfides. These findings suggest that the oxygen groups remain active and play a crucial role in suppressing polysulfide dissolution throughout extended cycling periods. Figure 6g presents X‐ray diffraction (XRD) patterns of the Li metal anode after 50 charge/discharge cycles. Cells with the High Ox‐SWCNT interlayer showed specific peaks at 36°, 52°, and 65°, corresponding to pristine Li metal. However, cells with both Low Ox‐SWCNT and pristine SWCNT interlayers exhibited additional peaks at 32°, 43°, 44°, 52° and 55° marked by purple arrows, attributed to Li_2_S_x_ phases indicating the formation of a passivation layer due to the diffusion of Li_2_S_x_.^[^ ^4^, ^40^ ^]^ This layer causes to interfacial resistance. Electrochemical impedance spectroscopy (EIS) analysis assessed the impact of oxygen functional groups on passivation layer formation and ionic conductivity. Figure 6h is the Nyquist plot of the negative imaginary versus real parts of the complex impedance for individual electrodes and electrochemical cells after the 100th discharge cycle. Li‐S cells with a pristine SWCNT interlayer exhibited higher interfacial resistance compared to those with Low Ox‐SWCNTs and High Ox‐SWCNTs, reflecting their inadequate suppression of the shuttle phenomenon. Consequently, our oxygen‐functionalized SWCNT interlayer significantly reduces resistance, indicating a potential enhancement in the cycle stability and ionic conductivity of Li–S batteries.

### 2.3 Ultra‐Flexible Li‐S Batteries with High‐Energy Density over 1 Ah

**Figure 7:**
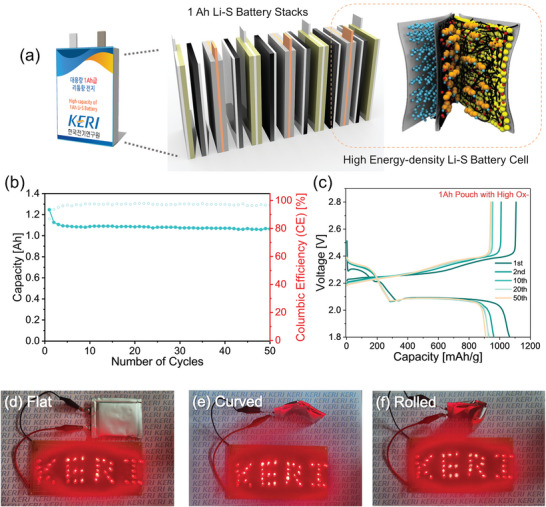
Electrochemical performance and flexible applications of the high‐capacity Li‐S pouch cells. a) Schematic illustrating the stacked structure of the 1 Ah Li‐S pouch cell. b) Discharge capacity (Ah) and Coulombic efficiency (CE) for the Li‐S pouch cells featuring CNT‐based sulfur cathodes and High Ox‐SWCNT interlayers. c) Voltage profiles for high‐capacity 1Ah Li‐S pouch cells over 50 cycles at a discharge rate of 0.1C. d–f) Photographs taken with a digital camera showing the LED lights powered by the flexible 1Ah Li‐S pouch cell in various deformation states: flat, curved, and rolled.

Finally, we developed large‐area and flexible Li‐S pouch cells with high electrochemical performance, consisting of a sulfur cathode combined with highly oxygen‐functionalized SWCNTs (High Ox‐SWCNTs) and a PE membrane incorporated with a High Ox‐SWCNT interlayer. As depicted in the schematic of **Figure** **7**a, this pouch‐type cell with high energy density comprises six stacks, each containing 10 cathodes and dimensions of 50 × 60 mm^2^. The cells feature a substantial active material loading of ≈39 mg cm^−2^, enabling an impressive initial discharge capacity of ≈1.25 Ah. Detailed calculations related to the pouch cell and information on each component are provided in the Supporting Information. Figure 7b,c illustrate the discharge capacities and voltage profiles of these high‐capacity Li‐S cells over 50 charge/discharge cycles. The cells maintained a capacity of 910 mAh g^−1^ over 50 cycles at a current density of ≈0.2 A cm^−^^2^. This performance demonstrates the successful integration of High Ox‐SWCNTs with sulfur electrodes and membranes, achieving both high energy density and the ability to sustain capacities over 1 Ah through 50 charge/discharge cycles. Despite their extensive area and multiple electrode stacks with interlayer‐coated membranes, our Li‐S cells exhibited stable and excellent electrochemical performance, maintaining minimal overvoltage, as shown in Figure 7c. We also demonstrated the successful operation of an LED module powered by our high‐capacity pouch cells, which exhibited ultra‐flexible mechanical properties and robustness against severe deformations such as curvature and rolling, achieved by eliminating the need for a metal current collector. Consequently, our high‐capacity, large‐area, and flexible Li‐S batteries are suitable for a wide range of innovative applications. Contrary to traditional rigid‐form batteries, our technology demonstrates broad applicability across multiple sectors, such as mobile phones, apparel, medical devices, smart textiles, flexible displays, electric vehicles, and urban air mobility (UAM) platforms. It offers high energy density and convenient portability owing to its flexible and free‐standing nature, thereby facilitating integration into a variety of shapes and sizes.

## 3 Conclusion

The large‐area and ultra‐flexible Li‐S batteries with high electrochemical performance were developed as next‐generation energy storage devices for diverse applications requiring free‐form factors and high energy density. These batteries consist of a free‐standing sulfur cathode and commercial membrane incorporating SWCNTs with oxygen‐containing functional groups. The scaffold‐based structure effectively suppresses the dissolution of LiPS by promoting Li‐O bond formation, with a higher content of oxygen functional groups absorbing more polysulfides. Furthermore, we introduced an Ox‐SWCNT interlayer coated on a commercial PE separator, where the porous structure of carbon and Li‐O bonds proved highly efficient in immobilizing polysulfides and mitigating shuttle effects. Consequently, our cells demonstrated significantly improved electrochemical performance, including high energy density, rapid redox kinetics, and excellent cycling stability, surpassing traditional Li‐S cells that use conventional carbon‐based nanomaterials and pristine PE membranes. We also successfully demonstrated a large‐size pouch‐type cell comprising six stacks, each containing 10 cathodes with dimensions of 50 × 60 mm^2^. The cells feature a substantial active material loading of ≈39 mg cm^−2^, enabling an impressive initial discharge capacity of ≈1.25 Ah. This performance reveals the successful integration of High Ox‐SWCNTs with sulfur electrodes and membranes, achieving both high energy density and the ability to sustain capacities over 1 Ah through extended charge‐discharge cycles. Unlike typical rigid‐form batteries, our technology offers high energy density and convenient portability due to its flexible and free‐standing nature, enabling integration into diverse shapes and sizes for broad applications through various sectors, including mobile smart devices, wearable medical devices, smart textiles, and long‐distance cruising electric vehicles.

## 4 Experimental Section

### Materials

Pristine SWCNT powder (TUBALL) produced via chemical vapor deposition was purchased from OCSiAl Asia Pacific Co., Ltd. For the oxidation and dispersion of the SWCNTs, sulfuric acid (DAEJUNG CHEMICALS & METALS), K_2_SO_4_ (Sigma‐Aldrich), and KMnO_4_ (JUNSEI) were used without further purification. Multi‐walled carbon nanotubes (MWCNTs, diameter = 9.5 nm) were purchased from Nanocyl (Belgium). Sulfur powder (99.98%), Li_2_S (99.98%), 1,3‐dioxolane (DOL, 99.8%), 1,2‐dimethoxyethane (DME, 99.5%), lithium bis(trifluoromethylsulfonyl)imide (LiTFSI, 99.95%), and lithium nitrate (LiNO_3_, 99.99%) were purchased from Sigma Aldrich (USA).

### Oxidation of the SWCNTs in the LC Phase

The SWCNTs were functionalized via an intercalation and oxidation process in H_2_SO_4_ (98%) media. Initially, 5 g of the SWCNT powder was mixed with 300 mL of H_2_SO_4_, and subsequently, 10 g of K_2_SO_4_ as an intercalant was added to the SWCNT/H_2_SO_4_ mixture under stirring. For the preparation of Low Ox‐SWCNTs, 2.5 g of KMnO_4_ was added slowly, and the mixture was stirred for 1 hour at room temperature, resulting in a lower density of oxygen‐functional groups. Conversely, for High Ox‐SWCNTs, 5 g of KMnO_4_ was added, and the mixture was stirred for 2 hours, achieving a higher density of oxygen‐functional groups due to increased oxidation. After the reaction, termination was implemented by adding a large amount of water, and the product was purified several times using centrifugation. The oxidized SWCNTs were then dispersed in NMP using a high‐pressure homogenizer (ATOMO 3.0, Beritoli) at 1000 bar with five cycles to obtain well‐dispersed SWCNT pastes without other dispersant molecules.

### Fabrication of CNT‐Based S Cathode

Sulfur powder was mixed with multi‐walled carbon nanotubes (Sulfur:MWCNT = 7:3  wt.%) in ethanol through ball‐milling (450 rpm, 30 min, 10 cycles). Ethanol was removed after vacuum filtration. The obtained MWCNT@S mixture was subsequently heated at 60 °C for 12 h in a vacuum oven to remove any trapped solvents and then heated at 155 °C for 2 h for melt‐diffusion of the elemental S into the CNT networks. The as‐prepared MWCNT@S was combined with SWCNT powder and CNT solutions (Pristine, Low Ox‐SWCNTs, or High Ox‐SWCNTs), in which the SWCNT powder was dispersed in H_2_O through ball‐milling for 10 min. This was followed by vacuum filtration and drying at 60 °C for 12 h in a vacuum oven. The CNT‐based S cathode was obtained with a controlled film thickness of 200 µm and a sulfur loading of ≈3 mg cm^−2^.

### Preparation of the Ox‐SWCNTs‐Based Interlayer

The interlayer was prepared using a mixture of SWCNT, SPB, and PVDF in a ratio of 5:2:3. This mixture was homogenized using a Thinky mixer with 10 mm balls and 5 mm balls, then cast onto a commercial PE separator at a thickness of 150 µm, and dried at 50 °C for 16 hours. The fabricated interlayer based on Ox‐SWCNTs has a thickness of ≈55 µm.

### Preparation of SWCNT/Li_2_S_8_ for Absorptivity Measurements

A 0.01 m Li_2_S_8_ solution was prepared by dissolving elemental S and Li_2_S in a molar ratio of 7:1 in DOL/DME at 50 °C with stirring for 24 h. Then, 10 mg pristine SWCNTs, Low Ox‐SWCNTs, and High Ox‐SWCNTs were added to 5 mL of DOL/DME solution, followed by the injection of an equal amount of Li_2_S_8_ solution to obtain the SWCNT/Li_2_S_8_ solution. All the procedures were performed in an argon‐filled glovebox.

### Process of Contact Angle Measurement

The contact angle of the interlayer was measured using a contact‐angle measurement system (Phoenix 300, SEO). Before the measurement, the goniometer was calibrated, and the needle position was adjusted so that a droplet just touched the surface of the sample. The needle's home and destination positions were fine‐tuned using the touch control settings. A 1 µL drop of 1 m LiTFSi in DOL/DME (1:1 v/v) + 1 wt.% LiNO₃ solution was deposited onto the interlayer surface using the “One drop”0 function. Images of the droplet were automatically captured by the system, and manual adjustments were made using the “Single” capture mode if necessary. The contact angle was automatically calculated by the software's analysis module, and in cases where automatic detection failed, a manual 2‐point method was used to define the droplet's baseline and apex.

### Electrochemical Characterization

Coin‐type (CR2032) cells were used for electrochemical characterization. A 100 µm of Li metal and a liquid electrolyte of 1 M lithium bis(trifluoromethylsulfonyl) imide (LiTFSI) were employed in DOL/DME (v/v = 1:1) with 1 wt.% LiNO_3_ as an additive. Li–S cell performance was investigated using a cycle tester (Won‐A Tech) at 25 °C under various charge/discharge conditions in the potential range of 1.8–2.8 V. The pouch‐type cell had specific sizes for the cathode (50×60 mm^2^), anode (52×62 mm^2^), and separator (55×65 mm^2^), with the anode consisting of a Li metal sheet with a thickness of 40 µm. An Al foil was attached to the side of the cathode with carbon paste, and a Cu foil was connected to the Li anode using a roll press machine. Lead tabs were provided by attaching an Al foil to the cathode and a Cu foil to the anode. The cell used a liquid electrolyte containing 1 M LiTFSI in DOL/DME = 1/1 (v/v) with a 1 wt.% LiNO_3_ additive, and the E/S ratio (ratio of electrolyte volume to S weight) was fixed at 10 mL g_s_^−1^ for all cells tested.

### Characterization

The thermal analysis of pristine and oxidized SWCNTs was implemented by TGA (TA Instruments, TGA Q500) in the air at a ramping rate of 10 °C min^−1^. The morphologies and microstructure of the materials were visualized by field‐emission scanning electron microscopy (FE‐SEM, GEMINI500, ZEISS) at 2 kV, and the energy dispersive X‐ray spectroscopy (EDS) was used to characterize the element distribution on the CNT‐based S cathode surface at 10 kV. X‐ray diffraction (XRD) patterns were measured using an X‐ray diffractometer (MiniFlex 600, Rigaku Corp., Japan). Diffraction data were collected in the 2θ range between 10° and 90° from a step size of 0.02°. After the sample was sealed and attached to a MiniFlex 600 diffractometer at 15 mA and 40 kV, X‐ray photoelectron spectra (XPS) were collected using a Nexsa (ThermoFisher Scientific) with an X‐ray spot size of 100 µm. The dissolution of Li_2_S_x_ in DOL/DME was analyzed using UV–vis absorption spectroscopy (V‐770, Jasco). Electrochemical impedance spectroscopy (EIS) data were collected with a VMP3 (Bio‐Logic) from 1 mHz to 1 MHz.

## Conflict of Interest

The authors declare no conflict of interest.

## Supporting information



Supporting Information

## Data Availability

The data that support the findings of this study are available from the corresponding author upon reasonable request.
