# Supplementary material for: A Promising Approach to Ultra‐Flexible 1 Ah Lithium–Sulfur Batteries Using Oxygen‐Functionalized Single‐Walled Carbon Nanotubes
Source: Adv Sci (Weinh). 2024 Dec 4;12(4):2406536. doi: 10.1002/advs.202406536 (PMC11775554; doi:10.1002/advs.202406536)
Supplement: Supplementary file 1 — Supporting Information [file ADVS-12-2406536-s001.docx]

**Supporting Information

A Promising Approach to Ultra-flexible 1 Ah Lithium-Sulfur Batteries Using Oxygen-Functionalized Single-Walled Carbon Nanotubes**

*Junyoung Heo^1,3^, Jeong-Won Hong^1^, Ha Won* *Gu^1^, Junghwan Sung^1,3^, Dong-Hee Kim^1,3^,
Jung Hoon Kim^2^, Sung Kang^5^, You-Jin Lee^1^, Hye Young Choi^1^, Doohun Kim^1,3^, Kang-Jun Baeg^4,*^,
Joong Tark Han^2,3,*^, Jun-Woo Park^1,3,*^*

^1^Next-Generation Battery Research Center, Korea Electrotechnology Research Institute (KERI), 12, Jeongiui-gil, Seongsan-gu, Changwon-si, Gyeongsangnam-do 51543, Republic of Korea

^2^Nano Hybrid Technology Research Center, Korea Electrotechnology Research Institute (KERI), 12, Jeongiui-gil, Seongsan-gu, Changwon-si, Gyeongsangnam-do 51543, Republic of Korea

^3^Department of Electro-Functionality Materials Engineering, University of Science and Technology (UST), 217, Gajeong-ro, Yuseong-gu, Daejeon 34113, Republic of Korea

^4^ Major of Semiconductor Engineering, Pukyong National University, 45, Yongso-ro, Nam-gu, Busan 48513, Republic of Korea

^5^ Analysis and Assessment Research Center, Research Institute of Industrial Science & Technology (RIST), 67 Cheongam-ro, Nam-gu, Pohang-si 37673, Republic of Korea

*Corresponding authors:

Jun-Woo Park: +82-55-280-1684/+82-55-280-1590. E-mail: parkjw@keri.re.kr

Joong Tark Han: +82-55-280-1678. E-mail: jthan@keri.re.kr

Kang-Jun Baeg: +82-51-629-6397. E-mail: kangjun100@pknu.ac.kr

The pouch-type cell had specific sizes for the cathode (50×60 mm^2^), anode (52×62 mm^2^), and separator (55×65 mm^2^), with the anode consisting of a Li metal sheet with a thickness of 40 µm. An Al foil was attached to the side of the cathode with carbon paste, and a Cu foil was connected to the Li anode using a roll press machine. Lead tabs were provided by attaching an Al foil to the cathode and a Cu foil to the anode. The cell used a liquid electrolyte containing 1M LiTFSI in DOL/DME = 1/1 (v/v) with a 1 wt.% LiNO_3_ additive, and the E/S ratio (ratio of electrolyte volume to S weight) was fixed at 10 mL g_s_^−1^ for all cells tested

1. **Energy Density and Calculation Method:**

- Energy density (excluding pouch):

$$Energy Density \left[ Wh/kg \right]= \frac{\left( Initial capacity \right)\times\left( Active material mass \right)\times\left( Average discharge voltage \right)}{\left( Cathode mass \right)+\left( Anode mass \right)+\left( Interlayer mass \right)+\left( Electrolyte mass \right)}$$

$$= \frac{1065.51\times1.17\times2.1}{2.25+0.60+0.10+5.54}=308[Wh/kg]$$

- Capacity [Ah]:

$$Capacity \left[ Ah \right]=Discharge capacity \left[ \frac{mAh}{g} \right] \times Active material mass \left[ g \right]$$

$=10665.51 \times1.17=1.25 [Ah]$

1. **Thickness and weigh of each component:**

| Component | Thickness[µm] | Weight[g] |
| --- | --- | --- |
| Al foil | 15 | 0.13 |
| Cu foil | 10 | 0.27 |
| Active material | - | 1.17 |
| Coated separator | 55 | 0.10 |
| Packing separator | 25 | 0.16 |
| Li metal | 40 | 0.6 |
| Electrolyte | - | 3.89 |
| Pouch | - | 3.33 |

**3. Method for Cathode Attachment to Al Foil:**

The cathode was attached to the Al foil using a carbon paste prepared by mixing SPB and PVDF in an 8:2 ratio. This mixture was cast onto the aluminum current collector, and then the cathode material was applied on top of the prepared surface.

**Figure S1**. Thermogravimetric analysis (TGA) profiles to quantify the oxidative functional groups of Pristine, Low-Ox, High Ox- SWCNT


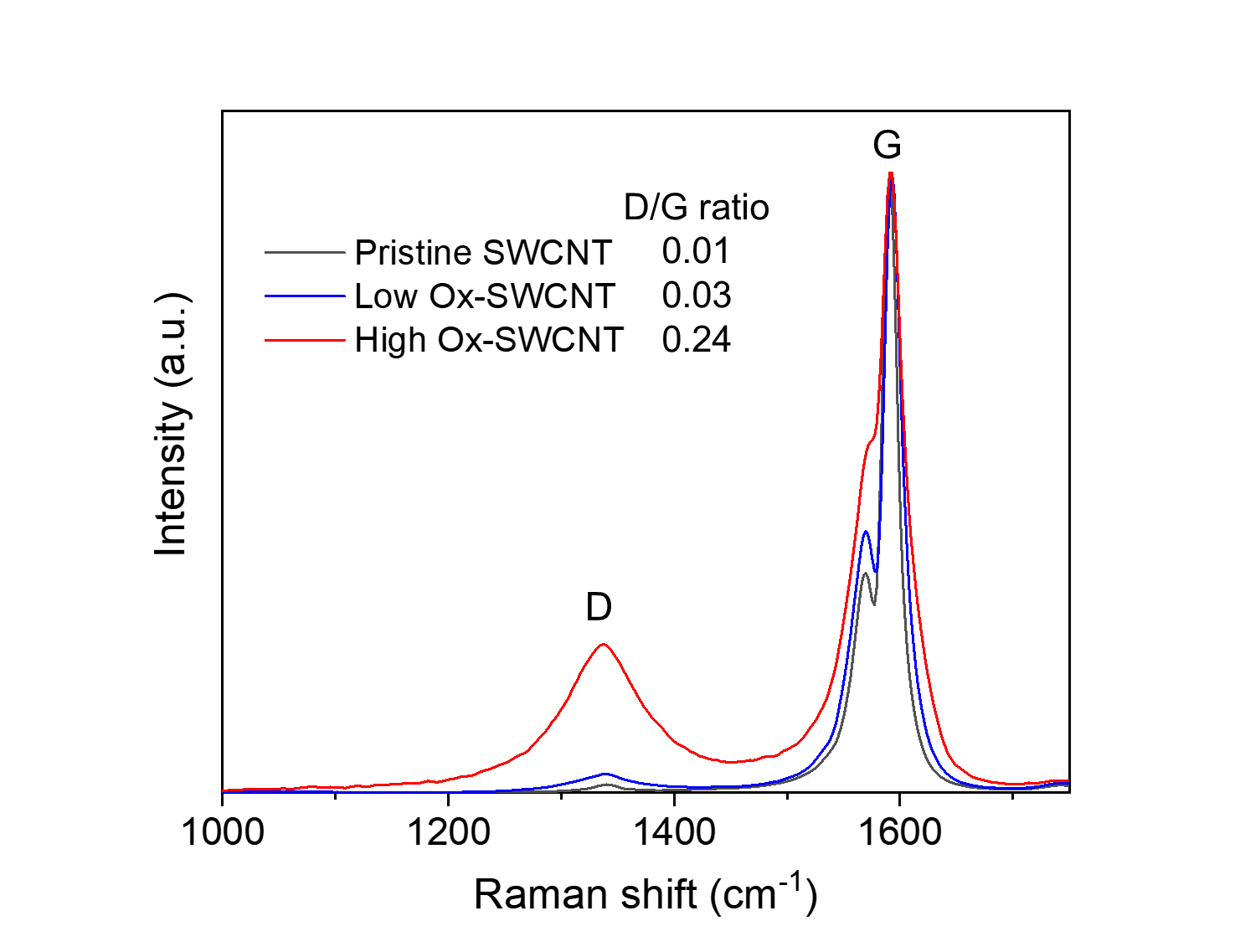
**Figure S2**. Raman Spectra of Pristine, Low and High Oxidation Level SWCNTs


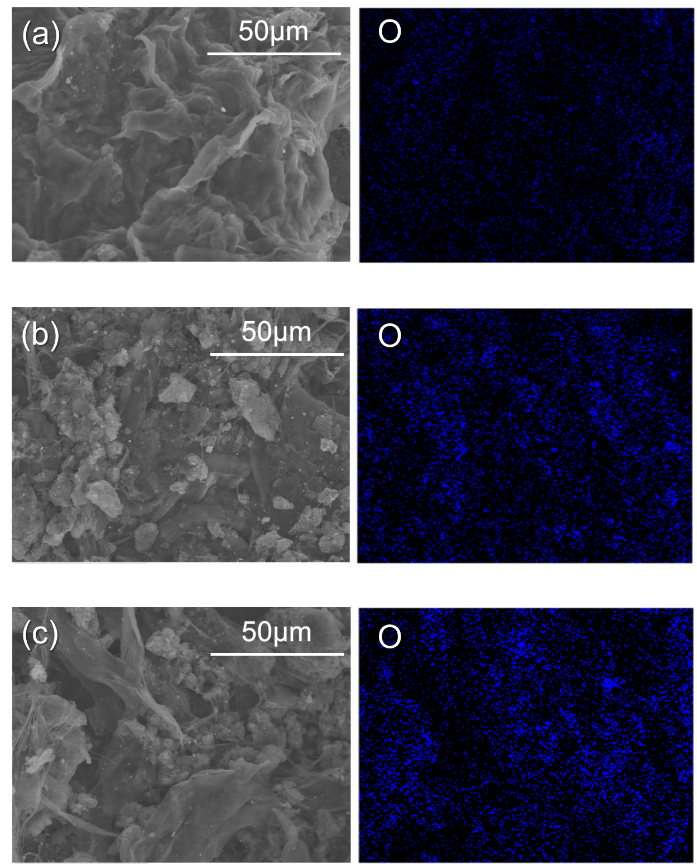


**Figure S3**. SEM images and EDS mapping of oxygen (O) element distribution for (a) Pristine, (b) Low Ox-SWCNT, and (c) High Ox-SWCNT samples.

**Table S1**. Atomic concentration in EDS mapping images of pristine, Low Ox-SWCNT and High Ox-SWCNT


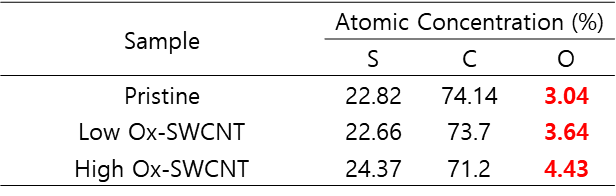


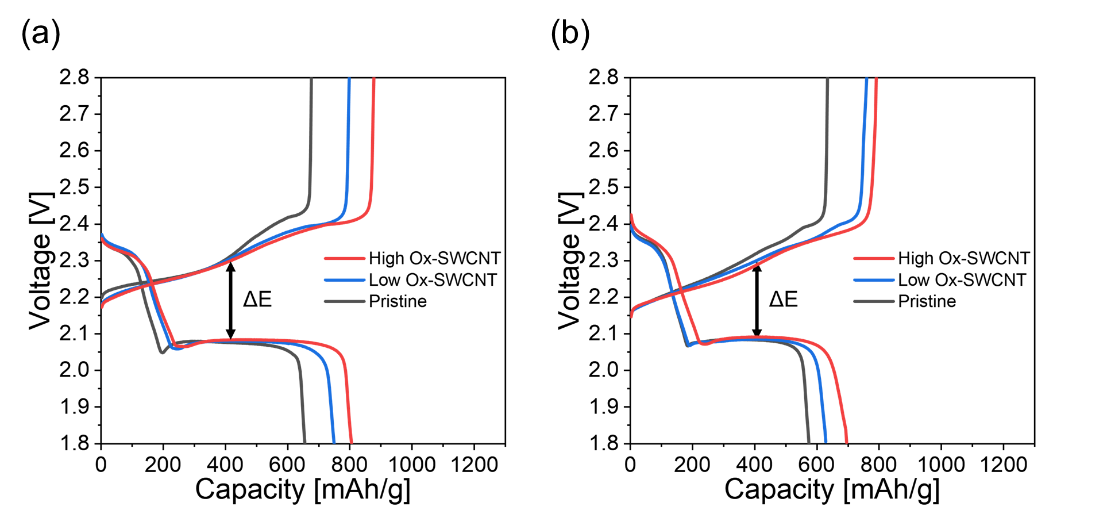


**Figure S4** Voltage profile of the second charge–discharge cycle for the (a) cathode and (b) separator using functionalized SWCNTs, illustrating the overpotential difference at 50% of total discharge capacity.


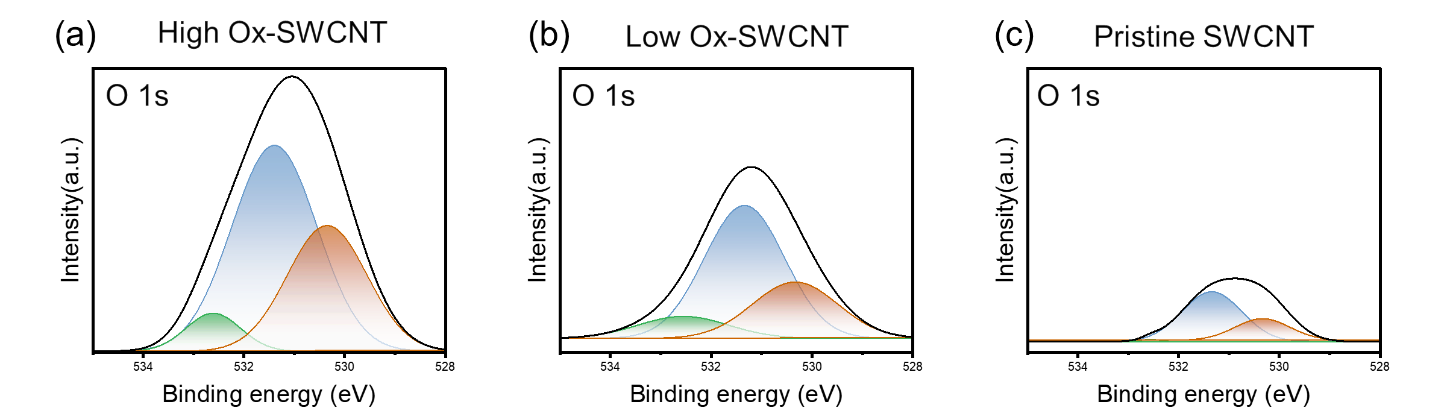


**Figure S5**. XPS O1s spectra of (a) High Ox-SWCNT, (b) Low Ox-SWCNT, and (c) Pristine cathodes after 50 cycles, indicating the presence of C=O, C-O, and Li-O bonds.


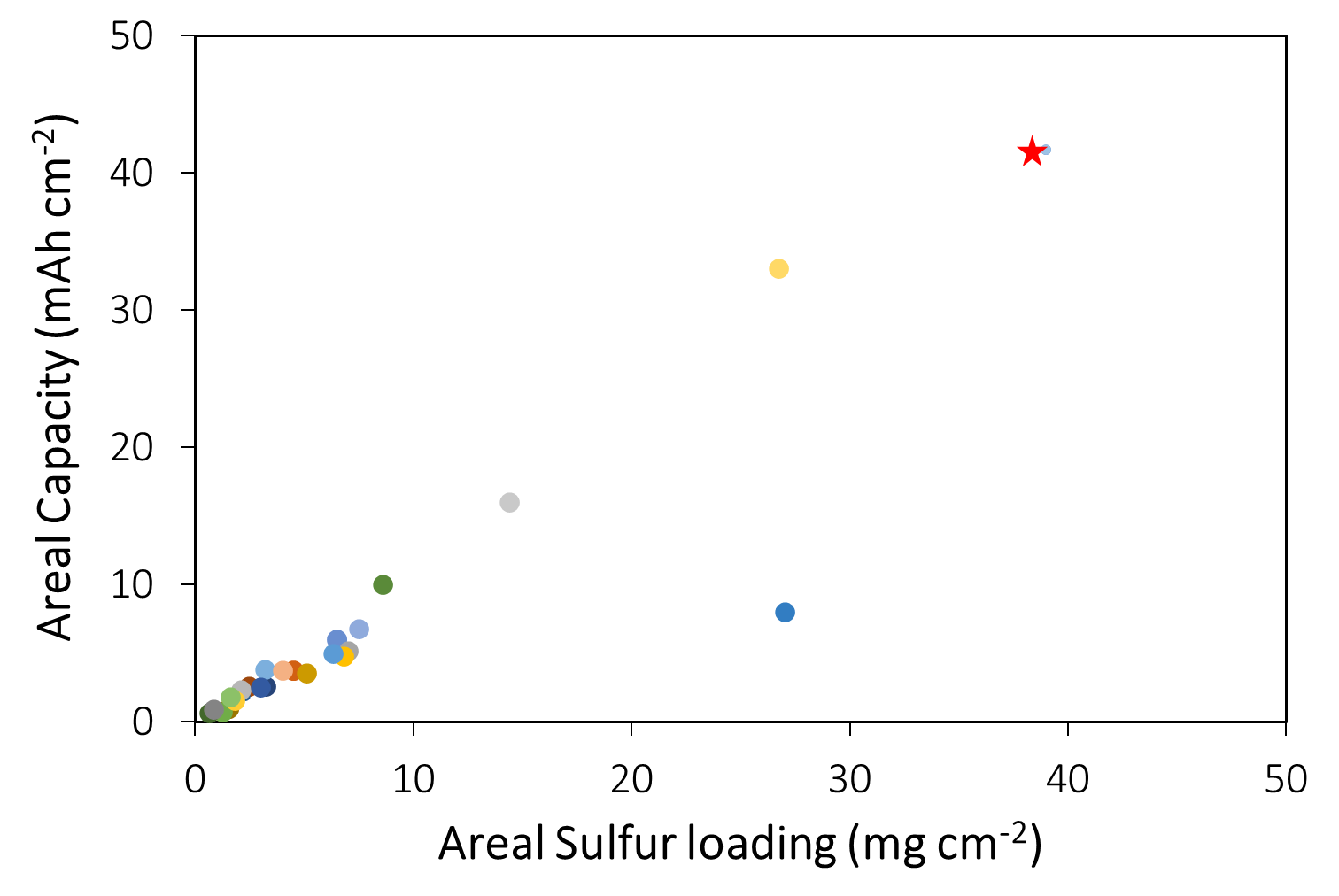


**Figure S6**. Areal Capacity of Li-S Cells as a Function of Areal Sulfur Loading for Flexible, Free-Standing High Ox-SWCNT-Based Sulfur Cathodes

**Table S2.** Performance comparisons between High OX- SWCNT based flexible sulfur cathode with representative self-standing and flexible sulfur cathodes.

| **References** | **C-rate** | **Areal Sulfur loading**  **[mg cm^-2^]** | **Areal Capacity**  **[mAh cm^-2^]** |
| --- | --- | --- | --- |
| **This Work** | **0.1C** | **39** | **41.67** |
| S1 | 0.2C | 2.5 | 2.2 |
| S2 | 0.1C | 7 | 5.2 |
| S3 | 0.5C | 6.8 | 4.8 |
| S4 | 0.1C | 6.3 | 5 |
| S5 | 0.9C | 1.25 | 0.71 |
| S6 | 0.1C | 3.25 | 2.6 |
| S7 | 0.18C | 2.45 | 2.57 |
| S8 | 0.1C | 1.53 | 0.95 |
| S9 | 1C | 2.3 | 1.9 |
| S10 | 0.1C | 6.5 | 6 |
| S11 | 0.5C | 2 | 2.2 |
| S12 | 0.06C | 0.63 | 0.63 |
| S13 | 0.2C | 3.9 | 3.86 |
| S14 | 0.2C | 2.1 | 2.3 |
| S15 | 0.9C | 1.8 | 1.53 |
| S16 | 0.2C | 3.2 | 3.84 |
| S17 | 0.25C | 1.6 | 1.84 |
| S18 | 0.5C | 3 | 2.55 |
| S19 | 0.2C | 4.5 | 3.74 |
| S20 | 0.2C | 0.83 | 0.91 |
| S21 | 0.2C | 5.1 | 3.57 |
| S22 | 0.2 mA/cm^2^ | 27 | 8 |
| S23 | 0.1C | 8.6 | 10 |
| S24 | 0.5 | 7.5 | 6.76 |
| S25 | 0.2C | 4 | 3.74 |
| S26 | 1 mA/cm^2^ | 14.4 | 16 |
| S27 | 1C | 26.75 | 33.07 |

**Referenes**

[S1]. J.-L. Shi, H.-J. Peng, L. Zhu, W. Zhu, Q. Zhang, Template growth of porous graphene microspheres on layered double oxide catalysts and their applications in lithium–sulfur batteries, Carbon 92, 96-105 (2015).

[S2]. X.-B. Cheng, H.-J. Peng, J.-Q. Huang, L. Zhu, S.-H. Yang, Y. Liu, H.-W. Zhang, W. Zhu, F. Wei, Q. Zhang, Three-dimensional aluminum foam/carbon nanotube scaffolds as long- and short-range electron pathways with improved sulfur loading for high energy density lithium-sulfur batteries, J. Power Sources 261, 264-270 (2014).

[S3] L. Li, Z. P. Wu, H. Sun, D. Chen, J. Gao, S. Suresh, P. Chow, C. V. Singh, N. Koratkar, A Foldable Lithium-Sulfur Battery, ACS Nano 9, 11342–11350 (2015).

[S4] Z. Yuan , H.-J. Peng , J.-Q. Huang, X.-Y. Liu, D.-W. Wang , X.-B. Cheng, Q. Zhang, Hierarchical Free-Standing Carbon-Nanotube Paper Electrodes with Ultrahigh Sulfur-Loading for Lithium–Sulfur Batteries, Adv. Funct. Mater. 24, 6105-6112 (2014).

[S5] G. Zhou, D.-W. Wang, F. Li, P.-X. Hou, L. Yin, C. Liu, G. Qing (Max) Lu, I. R. Gentle, H.-M. Cheng, A flexible nanostructured sulphur–carbon nanotube cathode with high rate performance for Li-S batteries Energy Environ. Sci. 5, 8901-8906 (2012).

[S6] K. Jin, X. Zhou, L. Zhang, X. Xin, G. Wang, Z. Liu, Sulfur/Carbon Nanotube Composite Film as a Flexible Cathode for Lithium-Sulfur Batteries J. Phys. Chem. C 117, 21112–21119 (2013).

[S7] G. Zhou, Design, Fabrication and Electrochemical Performance of Nanostructured Carbon Based Materials for High-Energy Lithium–Sulfur Batteries, Springer Theses (2017).

[S8] J. Jin, Z. Wen, G. Ma, Y. Lu, Y. Cui, M. Wu, X. Liang, X. Wu, Flexible self-supporting graphene–sulfur paper for lithium sulfur batteries, RSC Advances 3, 2558-2560 (2013).

[S9] J.-Q. Huang, H.-J. Peng, X.-Y. Liu, J.-Q. Nie, X.-B. Cheng, Q. Zhang, F. Wei, Flexible all-carbon interlinked nanoarchitectures as cathode scaffolds for high-rate lithium–sulfur batteries, J. Mater. Chem. A 2, 10869–10875 (2014).

[S10] R. Elazari , G. Salitra , A. Garsuch , A. Panchenko, D. Aurbach, Sulfur-Impregnated Activated Carbon Fiber Cloth as a Binder-Free Cathode for Rechargeable Li-S Batteries, Adv. Mater. 23, 5641-5644 (2011).

[S11] Z. Zhang, Q. Li, K. Zhang, W. Chen, Y. Lai, J. Li, Titanium-dioxide-grafted carbon paper with immobilized sulfur as a flexible free-standing cathode for superior lithium-sulfur batteries J. Power Sources 290, 159-167 (2015).

[S12] C. Wang, X. Wang, Y. Wang, J. Chen, H. Zhou, Y. Huang, Macroporous free-standing nano-sulfur/reduced graphene oxide paper as stable cathode for lithium-sulfur battery, Nano Energy 11, 678-686 (2015).

[S13] G. Zhou, Y. Zhao, A. Manthiram, Dual-Confined Flexible Sulfur Cathodes Encapsulated in Nitrogen-Doped Double-Shelled Hollow Carbon Spheres and Wrapped with Graphene for Li–S Batteries, Adv. Energy Mater. 5, 1402263 (2015).

[S14] C. Wu, L. Fu, J. Maier, Y. Yu, Free-Standing Graphene-based Porous Carbon Films with Three-Dimensional Hierarchical Architecture for Advanced Flexible Li-Sulfur Batteries, J. Mater. Chem. A 3, 9438-9445 (2015).

[S15] G. Zhou, L. Li, D.-W. Wang, X.-Y. Shan, S. Pei, F. Li, H.-M. Cheng, A Flexible Sulfur-Graphene-Polypropylene Separator Integrated Electrode for Advanced Li–S Batteries, Adv. Mater. 27, 641-647 (2015).

[S16] G. Zhou,Y. Zhao, C. Zu, A. Manthiramn, Free-standingTiO2 nanowire-embedded graphene hybrid membrane for advanced Li/dissolved polysulfide batteries, Nano Energy 12, 240-249 (2015).

[S17] H. Chen, C. Wang, Y. Dai, S. Qiu, J. Yang, W. Lu, L. Chen, Rational Design of Cathode Structure for High Rate Performance Lithium−Sulfur Batteries, Nano Lett. 15, 5443-5448 (2015).

[S18] H.-S. Kang, Y.-K. Sun, Freestanding Bilayer Carbon–Sulfur Cathode with Function of Entrapping Polysulfi de for High Performance Li–S Batteries, Adv. Funct. Mater. 26, 1225-1232 (2016).

[S19] W. Zhou, B. Guo, H. Gao, J. B. Goodenough, Low-Cost Higher Loading of a Sulfur Cathode, Adv. Energy Mater. 6, 1502059 (2016).

[S20] H. Wang, W. Zhang, H. Liu, Z. Guo, A Strategy for Configuration of an Integrated Flexible Sulfur Cathode for High-Performance Lithium–Sulfur Batteries, Angew. Chem. Int. Ed. 55, 3992-3996 (2016).

[S21] S.-H. Chung, Chi-Hao Chang, A. Manthiram, Robust, Ultra-Tough Flexible Cathodes for High-Energy Li–S Batteries, Small 12, 939-950 (2016).

[S22] Li, H., Feng, J., Liu, T., Qin, K., Zhu, X., & Suo, Suppressing sulfur crosstalk lowers the bar of lithium metal anode for practical Li-S pouch cells, Energy Storage Materials, 2024, 71: 103664.

[S23] Kim, H., Kim, J. M., Choi, H. N., Min, K. J., Kansara, S., Hwang, J. Y., ... & Sun, Y. K, Improving reaction uniformity of high‐loading lithium‐sulfur pouch batteries. Carbon Energy, 2024, e578.

[S24] Lee, J., Ko, B., Kang, J., Chung, Y., Kim, Y., Halim, W., ... & Joo, Y. L, Facile and scalable fabrication of highly loaded sulfur cathodes and lithium–sulfur pouch cells via air-controlled electrospray, Materials today energy, 6, 255-263.

[S25] Li, H., Wen, X., Shao, F., Zhou, C., Zhang, Y., Hu, N., & Wei, H, Interface covalent bonding endowing high-sulfur-loading paper cathode with robustness for energy-dense, compact and foldable lithium-sulfur batteries, Chemical Engineering Journal, 412, 128562.

[S26] Jiang, Z., Guo, H. J., Zeng, Z., Han, Z., Hu, W., Wen, R., & Xie, J, Reconfiguring organosulfur cathode by over-lithiation to enable ultrathick lithium metal anode toward practical lithium–sulfur batteries, ACS nano, 14(10), 13784-13793.

[S27] Wang, W., Xi, K., Li, B., Li, H., Liu, S., Wang, J., ... & Li, G, A sustainable multipurpose separator directed against the shuttle effect of polysulfides for high‐performance lithium–sulfur batteries,
